# Effect of Patient Portal Messaging Before Mailing Fecal Immunochemical Test Kit on Colorectal Cancer Screening Rates: A Randomized Clinical Trial

**DOI:** 10.1001/jamanetworkopen.2021.46863

**Published:** 2022-02-04

**Authors:** Gregory Goshgarian, Camille Sorourdi, Folasade P. May, Sitaram Vangala, Sarah Meshkat, Lily Roh, Maria A. Han, Daniel M. Croymans

**Affiliations:** 1 Department of Medicine, David Geffen School of Medicine, University of California, Los Angeles; 2 Department of Medicine, Central Michigan University College of Medicine, Mount Pleasant; 3 The Vatche and Tamar Manoukian Division of Digestive Diseases, Department of Medicine, David Geffen School of Medicine, University of California, Los Angeles; 4 Division of Gastroenterology, Greater Los Angeles Veterans Affairs Healthcare System, Los Angeles, California; 5 UCLA Kaiser Permanente Center for Health Equity, Jonsson Comprehensive Cancer Center, Los Angeles, California; 6 Department of Medicine Quality, University of California, Los Angeles, Los Angeles

## Abstract

**Importance:**

Colorectal cancer (CRC) screening reduces CRC mortality; however, screening rates remain well below the national benchmark of 80%.

**Objective:**

To determine whether an electronic primer message delivered through the patient portal increases the completion rate of CRC screening in a mailed fecal immunochemical test (FIT) outreach program.

**Design, Setting, and Participants:**

In this randomized clinical quality improvement trial at the University of California, Los Angeles Health of 2339 patients enrolled in a FIT mailing program from August 28, 2019, to September 20, 2020, patients were randomly assigned to either the control or intervention group, and the screening completion rate was measured at 6 months. Participants were average-risk managed care patients aged 50 to 75 years, with a valid mailing address, no mailed CRC outreach in the previous 6 months, and an active electronic health record (EHR) patient portal who were due for CRC screening. Data were analyzed on an intention-to-treat basis.

**Interventions:**

Eligible patients were randomly assigned to receive either (1) the standard FIT mailed outreach (control group) or (2) the standard FIT mailed outreach plus an automated primer to notify patients of the upcoming mailed FIT sent through the electronic patient portal (intervention group).

**Main Outcomes and Measures:**

The primary outcome was the screening completion rate (ie, returning the FIT). Secondary outcomes were (1) were the time to CRC screening from the FIT mailing date, (2) screening modality completed, and (3) the effect of opening the electronic primer on screening completion rate.

**Results:**

The study included 2339 patients (1346 women [57.5%]; mean [SD] age, 58.9 [7.5] years). The screening completion rate was higher in the intervention group than in the control group (37.6% [445 of 1182] vs 32.1% [371 of 1157]; *P* = .005). The time to screening was shorter in the intervention group than in the control group (adjusted hazard ratio, 1.24; 95% CI, 1.08-1.42; *P* = .003). The proportion of each screening test modality completed was similar in both groups. In a subanalysis of the 900 of 1182 patients (76.1%) in the intervention group who opened the patient portal primer message, there was a 7.3–percentage point (95% CI, 2.3-12.4 percentage points) increase in CRC screening (local mean treatment effect; *P* = .004).

**Conclusions and Relevance:**

Implementation of an electronic patient portal primer message in a mailed FIT outreach program led to a significant increase in CRC screening and improvement in the time to screening completion. The findings provide an evidence base for additional refinements to mailed FIT outreach quality improvement programs in large health systems.

**Trial Registration:**

ClinicalTrials.gov Identifier: NCT05115916

## Key Points

**Question:**

Does an electronic patient portal message primer increase the completion rate of colorectal cancer (CRC) screening in a mailed fecal immunochemical test (FIT) program?

**Findings:**

In this randomized clinical quality improvement trial of 2339 patients receiving a mailed FIT, the rate of screening completion was significantly higher in the group that received the electronic message than in the control group (38% vs 32%).

**Meaning:**

Including an electronic patient portal message primer prior to a mailed FIT is an effective method to increase CRC screening rates in a managed care patient population.

## Teaser

This randomized clinical trial examines whether an electronic primer message delivered through the patient portal increased colorectal cancer (CRC) screening in a mailed fecal immunochemical test outreach program.

## Introduction

Colorectal cancer (CRC) is the third most common cancer and the third leading cause of cancer-related deaths in the United States.^1,2^ Owing to the relatively slow growth of precancerous colon and rectal lesions, screening is effective for both prevention and early detection of disease and has been demonstrated to reduce mortality.^3,4^ The US Preventive Services Task Force recommends screening for all individuals from 50 to 75 years of age (grade A), and in May 2021, they updated their recommend screening guidelines to include individuals 45 to 49 years of age (grade B).^5,6,7^ Nonetheless, the US screening rate remains well below the national benchmark of 80% as established by the National Colorectal Cancer Roundtable.^1,8^

The fecal immunochemical test (FIT) is an effective screening modality for CRC.^9,10,11,12,13^ Although the US Preventive Services Task Force does indicate individuals complete screening via FIT, fecal occult blood test, stool DNA test, colonoscopy, computed tomography colonography, and flexible sigmoidoscopy, it does not recommend any one of the screening modality over another.^7^ The US Multi-Society Task Force, however, recommended FIT and colonoscopy as first-line screening modalities for CRC in 2017.^14^ This guidance has practical implications because FIT can be mailed to patients and completed at home, reaching patients who do not have access to colonoscopy or who have reservations about more invasive screening modalities. In light of decreasing CRC screening rates and patient hesitancy for in-person health visits during the COVID-19 pandemic, multiple medical and professional societies have emphasized the use of a mailed FIT outreach program.^15,16,17^

Mailed FIT outreach is effective at increasing CRC screening rates in health systems.^18,19,20,21,22,23,24^ The Community Preventive Services Task Force, in their 2012 systematic review, indicated with strong evidence that sending “client reminders or recalls” (ie, electronic or postal letters or telephone messages) increased screening completion rates via fecal occult blood test.^25^ Furthermore, a systematic review by Issaka et al^23^ demonstrated increased screening completion rates when mailed or telephone FIT primers were provided to patients prior to mailed FIT outreach. Primers preempt a future event (ie, arrival of a mailed FIT kit), with the goal to stimulate a positive behavioral response (ie, completion and return of the FIT kit), and are effective in improving health behaviors.^26,27,28,29,30^ Studies in behavioral economics and psychology indicate that how information or choice is framed affects behavior in predictable ways, which has applications in health and medicine, including the design of CRC screening strategies.^19,31,32,33,34^ However, while prior studies have demonstrated the positive effect of traditional mail or telephone primers by a median of 4.1%,^23^ there are no studies to date, to our knowledge that evaluate whether electronic primers can achieve the same gains. Leveraging electronic health record (EHR) patient portals to communicate with patients is a low-cost alternative to what has previously been demonstrated to increase CRC screening.

Thus, we developed an electronic primer within the EHR patient portal to alert patients due for CRC screening before arrival of a mailed FIT kit.^35,36,37^ As a part of our institution’s ongoing quality improvement efforts to increase CRC screening rates, we randomized implementation of the primer at the patient level to determine whether the electronic primer increased the CRC screening completion rate among patients enrolled in our mailed FIT program.

## Methods

### Setting and Population

This randomized clinical quality improvement trial was conducted from August 28, 2019, to September 20, 2020 (trial protocol in Supplement 1). University of California, Los Angeles (UCLA) Health is a large, integrated academic health system, with more than 390 000 primary care patients and 180 ambulatory primary care clinics across Southern California. Approximately 68 000 of these primary care patients form the managed care population, an insured group of patients enrolled in commercial health maintenance organization contracts covering services delivered only via UCLA Medical Group. In 2015, we implemented a biannual mailed FIT outreach program for managed care primary care patients who were overdue for average-risk CRC screening.^20^ All individuals between the ages of 50 and 75 years in this population who were overdue for CRC screening, based on their EHR information and the National Committee for Quality Assurance Healthcare Effectiveness Data and Information Set criteria, received a mailed FIT.^20,38^ The UCLA institutional review board deemed our work exempt because it is systems improvement consistent with ongoing hospital quality improvement efforts; patient consent was also waived because the study pertains to ongoing hospital quality improvement efforts. Our institution is implementing randomization as a best practice for quality improvement projects. As such, in reporting these findings to the public, we adhere to the standards set forth by the scientific community when randomizing participants to treatment. In addition, for this investigation, we followed and adhered to the Standards for Quality Improvement Reporting Excellence (SQUIRE) 2.0 reporting guideline for quality improvement projects and the Consolidated Standards of Reporting Trials (CONSORT) reporting guideline for randomized clinical trials.^39,40^

The FIT outreach mailing includes an informational letter, which uses principles of behavioral science, to offer CRC screening (eAppendix 1 in Supplement 2), a preaddressed OC-Auto FIT CHEK Kit (Polymedco Inc) with instructions, and a 1-page form to report non-UCLA screening results.^19^ The letter accompanying the FIT kit provided the option to schedule a colonoscopy if preferred. All FIT kit mailers contained a postal label for return to the UCLA clinical laboratory and were processed per manufacturer protocol. For positive results, a population health patient coordinator notified the patient’s primary care physician of the result, and the primary care physician was responsible for generating a referral for a diagnostic colonoscopy.

### Patient Selection and Randomization

With assistance from the UCLA Office of Population Health and Accountable Care, we used EHR data, billing data, and claims data to identify managed care patients, aged 50 to 75 years, at average risk for CRC and due for screening in August 2019 and again in March 2020. Due for screening was defined by a lack of FIT within the past year, flexible sigmoidoscopy within the past 5 years, colonoscopy within the past 10 years, computed tomography colonography within the past 5 years, or FIT-DNA test within the past 3 years. We excluded patients who had an invalid mailing address, had an inactive EHR patient portal (UCLA Health uses Epic MyChart), or who were considered high risk for CRC (personal history of CRC, adenomatous polyps, inflammatory bowel disease, familial polyposis syndrome, or a family history of CRC). We also excluded patients from inclusion to the March 2020 mailed outreach program who received a FIT mailer within the past 6 months.

In August 2019 and March 2020, we used simple randomization using a random number generator to assign eligible patients to receive either (1) the standard mailed FIT kit (control group) or (2) the standard mailed FIT kit plus an automated electronic primer sent through the personal health portal, alerting them that a FIT kit would be arriving to their home (intervention group).

### Intervention Description

Only individuals with activated EHR patient portals were included in the investigation. Both patient groups received identical informational letters and FIT kits in the outreach (eAppendix 1 in Supplement 2). In addition to this mailed outreach, patients in the intervention group received an electronic message through their personal health portal (eAppendix 2 in Supplement 2). Patients received the primer approximately 1 to 2 weeks prior to arrival of the FIT kit, which informed patients about the incoming FIT kit and instructed patients to complete and return the kit promptly.

### Outcomes and Variables

Our primary outcome was the CRC screening completion rate from FIT mailing date, over a 6-month follow-up period. We considered patients as screened for CRC if any screening modality (FIT, flexible sigmoidoscopy, colonoscopy, computed tomography colonography, or FIT-DNA) was completed between August 28, 2019, and February 28, 2020, for the August 2019 mailing and between March 20 and September 20, 2020, for the March 2020 mailing. We had 3 secondary outcomes: (1) time to screening completion in each group; (2) screening modality completion in each group at 6 months; and (3) receipt of the primer in the intervention group, as measured by opened primer message in the patient portal. We also included patient-level demographic variables, including age at time of randomization, sex, and race and ethnicity. The outcomes for this investigation were registered retrospectively.

### Statistical Analysis

For all analyses, we excluded patients who died during the study period. We also excluded patients from the August 2019 cohort from analysis who received a mailed FIT kit in the previous 6 months, eliminating potential confounding among patients who received a FIT kit twice during the study period. We used frequencies and mean and median values to summarize demographic data for each study group. We first compared screening completion rates in the 2 study groups using an intention-to-treat analysis and the *t* test. We then used logistic regression and Cox proportional hazards regression model to compare time to screening in the 2 study groups, controlling for age, sex, and race and ethnicity; results were presented with odds ratios or hazards ratios and 95% CIs. After this, we used the Fisher exact test to compare the completion rates of individual screening modalities in the 2 study groups. Last, we performed a secondary analysis to determine the effect of opening the portal message on screening, using randomization group as an instrumental variable. In this analysis, we compared the subset of patients in the intervention group who opened the portal primer message with the control group. All *P* values were from 2-sided tests and results were deemed statistically significant at *P* < .05. All analyses were performed using R, version 3.6.2 (R Group for Statistical Computing).^41^ All analyses performed, despite formal registration occurring during manuscript development, were developed and conducted a priori.

## Results

### Characteristics of the Study Population

**Figure 1.**
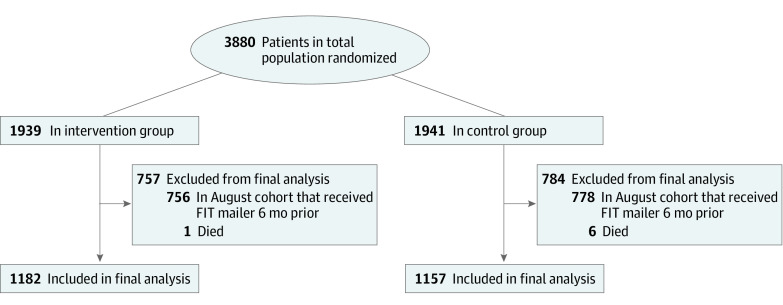
Patient Flow Diagram FIT indicates fecal immunohistochemical test.

There were 1157 patients (666 women [57.6%]; mean [SD] age, 58.7 [7.5] years) in the control group and 1182 patients (680 women [57.5%]; mean [SD] age, 59.2 [7.5] years) in the intervention group (Figure 1 and Table 1). The sample included 346 Hispanic individuals (14.8%), 1350 White individuals (57.7%), and 196 Black individuals (8.4%). These baseline patient characteristics were similar in both groups.

**Table 1.** Sample Characteristics^a^

Characteristic	Patients, No. (%)	
	Intervention (n = 1182)	Control (n = 1157)
Age, mean (SD), y	59.2 (7.5)	58.7 (7.5)
Median (IQR), y	58 (52-65)	57 (52-65)
Range, y	50-75	50-75
Sex		
Female	680 (57.5)	666 (57.6)
Male	502 (42.5)	491 (42.4)
Ethnicity		
Hispanic	182 (15.4)	164 (14.2)
Non-Hispanic	896 (75.8)	901 (77.9)
Unknown	104 (8.8)	92 (8.0)
Race		
American Indian or Alaska Native	1 (0.08)	0
Asian	133 (11.3)	146 (12.6)
Black	98 (8.3)	98 (8.5)
White	681 (57.6)	669 (57.8)
Other^b^	172 (14.6)	172 (14.9)
Unknown	92 (7.8)	69 (6.0)

^a^ This information was obtained from the electronic health records.

^b^ All other races that a person can identify as outside of American Indian or Alaska Native, Asian, Black, White, or unknown. These categories were determined at the system level.

### CRC Screening Completion Rates

At 6 months, the screening completion rate was 32.1% (n = 371) in the control group and 37.6% (n = 445) in the intervention group (*P* = .005) (Table 2). After adjusting for patient demographic characteristics, we found that the intervention group had significantly increased odds of completing CRC screening compared with the control group (odds ratio, 1.29; 95% CI, 1.08-1.53; *P* = .004) (eTable 1 in Supplement 2).

**Table 2.** Colorectal Cancer Screening Completion Rate at 6 Months After Intervention

Study group	Patients, No. (%)		*P* value^a^
	Not up to date	Up to date	
Intervention (n = 1182)	737 (62.4)	445 (37.6)	.005
Control (n = 1157)	786 (67.9)	371 (32.1)	

^a^ Significance set at *P* < .05.

**Figure 2.**
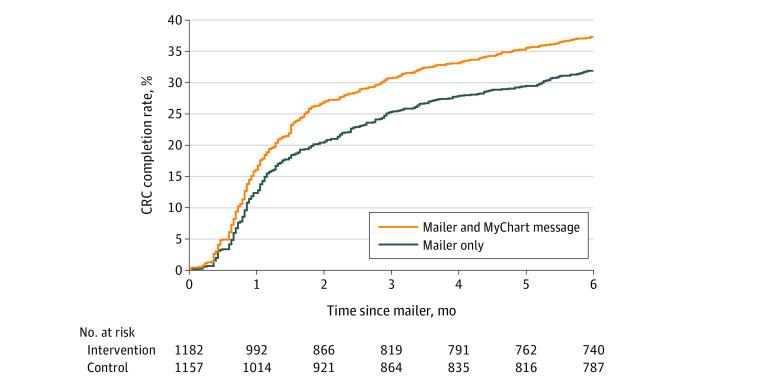
Cumulative Risk Curve, Stratified by Study Group CRC indicates colorectal cancer.

Furthermore, Figure 2 displays the estimated cumulative incidence of CRC screening for each group, showing that the intervention group diverged from the control group in the first 2 months of the intervention, after which it maintained consistently higher screening rates. The median time to CRC screening completion after receipt of the mailer among those who received the FIT kit within the 6-month follow-up was 35 days (IQR, 22-72 days) for the intervention group and 38 days (IQR, 24-84 days) for the control group. The Cox proportional hazards regression model demonstrated that the time to CRC screening in the intervention group was significantly shorter than the time to screening in the control group (hazards ratio, 1.24; 95% CI, 1.08-1.42; *P* = .003) (Table 3).

**Table 3.** Cox Proportional Hazards Regression Model

Effect	Adjusted HR (95% CI)	*P* value^a^
Intervention vs control	1.24 (1.08 to 1.42)	.003
Age (per 1-y increment)	1.02 (1.01 to 1.03)	<.001
Male vs female	1.14 (0.99 to 1.31)	.06
Ethnicity		
Hispanic	0.88 (0.70 to 1.10)	.25
Non-Hispanic	1 [Reference]	NA
Unknown	1.48 (1.08 to 2.02)	.02
Race		
American Indian or Alaska Native	<0.01 (<0.01 to >0.99)	.99
Asian	1.25 (1.02 to 1.54)	.03
Black	1.01 (0.78 to 1.30)	.96
White	1 [Reference]	NA
Other	1.02 (0.82 to 1.26)	.89
Unknown	0.62 (0.42 to 0.91)	.02

Abbreviations: HR, hazard ratio; NA, not applicable.

^a^ Significance set at *P* < .05.

Furthermore, for both our adjusted logistic regression and Cox proportional hazards regression analysis, age and Asian race were significantly associated with CRC screening (age: hazard ratio, 1.02; 95% CI, 1.10-1.03; *P* < .001; Asian race: hazard ratio, 1.25; 95% CI, 1.02-1.54; *P* = .03) (eTable 1 in Supplement 2; Table 3). In addition, unknown race and ethnicity were also significantly associated with CRC screening (hazard ratio, 0.62; 95% CI, 0.42-0.91; *P* = .02); however, these variables need to be interpreted with caution (eTable 1 in Supplement 2; Table 3).

### Screening Modality Used

Screening modalities did not differ between the study groups (*P* = .65) (eTable 2 in Supplement 2). For the 371 patients in the control group who completed screening, 337 (90.8%) used FIT and 34 (9.2%) underwent colonoscopy; no patients underwent flexible sigmoidoscopy. For the 445 patients in the intervention group who completed screening, 409 (91.9%) used FIT, 35 (7.9%) underwent colonoscopy, and 1 (0.2%) underwent flexible sigmoidoscopy.

### Receipt of Patient Portal Primer

A total of 900 patients (76.1%) in the intervention group opened the patient portal primer message. We found that those who opened the message had a 7.3–percentage point increase (95% CI, 2.3-12.4 percentage points) in CRC completion (local mean treatment effect; *P* = .004).

## Discussion

In our study, we found that the addition of an electronic primer message sent through patients’ EHR portal significantly increased the CRC screening completion rate by 5.5% for patients in our mailed FIT outreach program. The rate of screening completion was statistically significant among patients who opened the primer message. The effect of the electronic primer was most prominent during the first month after mailing and was maintained throughout the 6-month study period despite an attenuation in screening participation over time. Screening test choice did not vary between the study groups. We believe that our findings highlight the growing importance of early detection in CRC prevention and treatment, as well as the growing need for effective and scalable screening programs.

This investigation has been a part of a series of quality improvement projects at UCLA Health focused on increasing CRC screening rates through a mailed FIT outreach program.^19,20^ Our intervention group had higher screening rates (37.6%) than previously published studies in the same managed care population (24.1% in the study by Bakr et al^19^ and 33.2% in the study by Yu et al^20^). We believe the increased screening completion rates in our study is due to improvement in care processes that have occurred in the years since the FIT outreach program began, the primer implementation, and the health care system’s emphasis on increasing CRC rates for this population. The absolute effect size (5.5%-7.3%) of our intervention on CRC screening completion rates was similar to other studies that have evaluated the effect of primers on FIT outreach.^42,43,44,45^ In an analysis of a mailed FIT outreach program in a large health maintenance organization in Colorado, use of a telephone primer increased the screening completion rate by 4.5%.^45^ In a separate population-based Dutch randomized clinical trial that used a similar mailed letter primer design as ours, researchers saw a 3.3% increase in the screening completion rate.^42^

Our findings are also consistent with behavioral science literature that demonstrates that successful implementation of priming messages influences behavior. Primers motivate individual behavior by providing a stimulus that will influence action on a subsequent process, using subconscious memory association.^26,27,46^ Although there is debate about the robustness of primers, primer messages have been used successfully in psychology to influence task completion, biases, and health behaviors.^27,28,30,47,48^ Specifically, primers have been shown to be effective at improving health behaviors, such as increasing physical activity, vaccination uptake, reducing unhealthy snack purchase, and increasing hand hygiene in clinical situations.^27,28,36,37,49^

### Strengths and Limitations

Our study has some strengths. First, the randomization successfully resulted in balanced study groups, which is the optimal way to control for potential unmeasured confounders. Second, our patient population is racially and ethnically diverse, which enhances the generalizability of our findings to other study populations. Third, although existing studies have demonstrated the benefits of primers delivered via telephone, this is the first study, to our knowledge, to show the benefit of an electronic primer in conjunction with a mailed FIT outreach program. Fourth, our intervention is low cost and does not require additional mailings or personnel, so it can be easily adapted for any health care system that integrates patient communication electronically.

Our study also has some limitations. First, our investigation was conducted in a single academic health system, which may limit the generalizability of the findings. Our health system is large, however, and the patient population is diverse. Second, we included and randomized only patients with active patient EHR portal accounts, which introduces the possibility of selection bias. Our findings may be more relevant to patient populations more engaged in their health or who are more technologically savvy. In our health care system, approximately 84% of patients have an activated portal, so this population is quite robust. Third, the population under investigation is routinely included in CRC screening efforts by the health care system. Although our randomization was balanced, a potential limitation of our analysis is that we did not adjust for whether patients received or completed prior CRC screening. Fourth, by focusing only on individuals who had not received FIT in the prior 6 months, we omitted individuals who may be particularly difficult to screen. Omitting these patients was necessary to create comparable populations and pool the August 2019 and March 2020 mailed outreach cohorts.

## Conclusions

Our randomized clinical quality improvement study in a large academic health care system demonstrated that a simple electronic patient portal primer sent prior to mailed FIT outreach may significantly increase CRC screening rates. Our future work will focus on understanding populations that do not participate in screening despite our current efforts and the design of interventions to reach these populations. Increasing suboptimal CRC screening rates in the US is essential to reducing the burden of CRC, and using simple, low-cost, and effective population health interventions, grounded in proven behavioral principles, will allow us to detect disease earlier and improve the quality of life of our patients.

## Notes

Supplement 1.
Trial Protocol

Supplement 2. **eAppendix 1.** FIT Kit Mailer Letter **eAppendix 2.** MyChart Electronic Message Example **eTable 1.** Logistic Regression Model of CRC Screening by Study Group **eTable 2.** Screening Modality by Study Group

Supplement 3.
Data Sharing Statement
