# Supplementary material for: Effect of Patient Portal Messaging Before Mailing Fecal Immunochemical Test Kit on Colorectal Cancer Screening Rates: A Randomized Clinical Trial
Source: JAMA Netw Open. 2022 Feb 4;5(2):e2146863. doi: 10.1001/jamanetworkopen.2021.46863 (PMC8817202; doi:10.1001/jamanetworkopen.2021.46863)
Supplement: Supplement 1. — Trial Protocol [file jamanetwopen-e2146863-s001.pdf]

Protocol: Effect of Patient Portal Messaging Before Mailing Fecal Immunochemical Testing on Colorectal Cancer Screening Rates

Importance: Colorectal Cancer screening rates remain below ideal levels as set forth by the National Colorectal Cancer Roundtable. Additionally, the screening rates at our institution mimic these national trends. As a routinely top 3 leading cause of cancer, and cancer related deaths in the USA, optimizing colorectal cancer (CRC) screening is a priority of our health system to prevent undue morbidity and mortality.

Location: UCLA Health, Los Angeles, California, USA, 90095

Population: 3880 individuals within in the UCLA Medical Group, a managed care population group, 50-75 years old, who are due or overdue for average risk colorectal cancer screening as defined by lack of FIT within the past year, Flexible sigmoidoscopy within 5 years, colonoscopy within 10 years, CT colonography within 5 years, or FIT-DNA within 3 years.

Study design:
Randomization: Patients were randomized into the study at two time intervals, August 2019 and March 2020. FIT Kits were sent on August 28, 2019 and March 28, 2020 and each cohort was followed for 6 months.
Exclusion Criteria: Patients were excluded if they did not have a valid mailing address, activated MyChart electronic patient portal, or were considered high risk for CRC (personal history of CRC, adenomatous polyps, IBD, familial polyposis syndrome, or family history of CRC). In the March 2020 cohort, individuals were excluded from randomization if they had been randomized into the August 2019 cohort, or received FIT within 6 months.
Intervention: There were two study populations. Standard FIT/Control group will receive our standard FIT mailing outreach which includes an introductory letter outlining the importance of screening, pre addressed PolymedCo OC-Auto FIT CHEK KIT with instructions, and a 1 page form to report non-UCLA CRC-related screening (e.g. colonoscopy completed outside of UCLA Health). The Intervention Group included the standard mailed FIT KIT along with an electronic priming message sent to their electronic health record (EHR) health portal informing them of an incoming FIT Kit and recommendations to get screened.
Outcome: Primary - CRC screening completion from FIT mailing date, over a 6 month follow-up, via any CRC screening modality. Secondary – 1) time to screening completion in each arm, 2) screening modality completion in each arm at 6 months, and 3) screening completion rates adjusted by receipt of the primer in the intervention arm, as measured by opened primer message in the EHR patient portal

Analysis Plan: All analyses were discussed *a-priori*, but were not formally submitted to the IRB for research purposes because our work was deemed exempt by our IRB under Quality Improvement provision. Our institution is implementing randomization within its quality improvement projects as a standard practice to help improve reliability of results. Participants were excluded from analysis if they died during the study period, and in the August 2019 cohort if they received a FIT mailer 6 months prior. We utilized descriptive statistics to summarize our data. An intention-to-treat analysis was utilized. Significance was set at values of less than 0.05. We first compared screening completion rates using t-tests. We then utilized logistic regression, and Cox proportional hazard controlling for age, sex, race and ethnicity to evaluate for screening completion rates and time to screening between the groups, and utilizing appropriate measures of association including odds ratios, hazard ratios, and 95% CI. To compare screening completion from individual modalities, we used Fisher exact test. To determine the impact of opening the portal message on screening completion we used the randomization arm as an instrumental variable, where we compared the subset of patients in the intervention group that opened the primer to the control group.

Registration: Clinical trials submission was completed after analysis, and in preparation of submission for publication. All procedures and analysis were discussed *a-priori*, and no analyses were completed *post hoc*. Identification number - NCT05115916.
