# Supplementary material for: Effect of Patient Portal Messaging Before Mailing Fecal Immunochemical Test Kit on Colorectal Cancer Screening Rates: A Randomized Clinical Trial
Source: JAMA Netw Open. 2022 Feb 4;5(2):e2146863. doi: 10.1001/jamanetworkopen.2021.46863 (PMC8817202; doi:10.1001/jamanetworkopen.2021.46863)
Supplement: Supplement 2. — eAppendix 1. FIT Kit Mailer Letter eAppendix 2. MyChart Electronic Message Example eTable 1. Logistic Regression Model of CRC Screening by Study Group eTable 2. Screening Modality by Study Group [file jamanetwopen-e2146863-s002.pdf]

Supplementary Online Content

Goshgarian G, Sorourdi C, May FP, et al. Effect of patient portal messaging before mailing fecal immunochemical test kit on colorectal cancer screening rates: a randomized clinical trial. *JAMA Netw Open.* 2022;5(2):e2146863. doi:10.1001/jamanetworkopen.2021.46863

eAppendix 1. FIT Kit Mailer Letter
eAppendix 2. MyChart Electronic Message Example
eTable 1. Logistic Regression Model of CRC Screening by Study Group
eTable 2. Screening Modality by Study Group

This supplementary material has been provided by the authors to give readers additional information about their work.

© 2022 Goshgarian G et al. *JAMA Network Open.*

eAppendix 1. FIT Kit Mailer Letter

UCLA MRN: «mrn»

Primary Care Doctor: «provider»

«fnm» «lnm»
«ADD_LINE_1» «ADD_LINE_2»
«CITY», «STATE_C» «ZIP»

August 2019

Screening Saves Lives!
Don't delay. Take the test and send it back today!

Dear «fnm» «lnm»,

There is an **easy, effective, and often free** colon cancer screening test called the **FIT Kit** that can find signs of **colon cancer** before you have symptoms. This **highly-effective** test can be done at home and can save your life. UCLA health and your primary care physician recommend that you complete this test because you are between the ages of 50 to 75 and may be due for colon cancer screening.

Did you know that **colon cancer is the 2nd leading cause of cancer deaths** in the USA? Completing this test, and sending it back to your UCLA Health care team, will help us screen you for early signs of colon cancer, which often presents with little or no symptoms. However, with **early detection**, through this FIT Kit, colon cancer can be **cured 9 out of 10 times.**

The FIT Kit is a preferred test by many people for colon cancer screening. Your actions today make the biggest difference! Catching colon cancer as soon as possible is your best chance for successful treatment. Enclosed is your FIT Kit test. **Do the test at home and send it back to us.**

Let's work towards a healthier you!

© 2022 Goshgarian G et al. *JAMA Network Open.*

For the test:

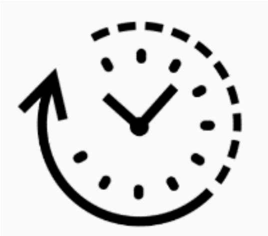

Screening takes minutes at home. Simply follow the directions and mail back your sample in the **pre-paid Business Envelope addressed to the UCLA-BURL/Pathology Outreach Services.**

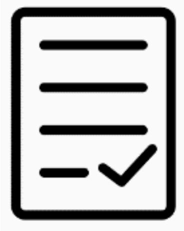

Get results fast. Be sure to return the completed, enclosed FIT Kit right after collecting your sample! Your primary care physician will have your results 2 weeks after you mail back your sample.

If you would prefer to schedule a colonoscopy procedure, as opposed to completing the FIT Kit, please contact your primary care physician.

If you have already completed your colon cancer screening, please help us update our records by filling out the next page.

Wishing you a year of health,

Samuel A. Skootsky, MD
Chief Medical Officer
Faculty Practice Group & UCLA Medical Group
UCLA Health

© 2022 Goshgarian G et al. *JAMA Network Open.*

eAppendix 2. MyChart Electronic Message Example

Final Subject line: A Message From Your Doctor
Content: Below

Screening Saves Lives!
Don't Delay. Take Your Test and Send it Back Today!

Dear «first name» «last name»,

Our records indicate **you are due for colon cancer screening**. **UCLA Health and your primary care doctor are mailing you** an easy, effective and often free colon cancer screening test called **a FIT Kit within the next week.**

Once you receive your FIT Kit, please complete, and send it back. This will help us screen you for early signs of colon cancer, which often presents with little or no symptoms. **Let's work together towards a healthier you!**

If you have any questions, or do not receive a FIT Kit in the next 2-3 weeks and would like one mailed to you, please contact your primary care doctor to receive your test.

Wishing you a year of health,

Samuel A. Skootsky, MD
Chief Medical Officer
Faculty Practice Group & UCLA Medical Group
UCLA Health

For more information on FIT Kit's and colon cancer screening please follow the links below.

- FIT Kit Educational website: https://www.uclahealth.org/colon-cancer-screening/fit
- Fit Kit Informational video: https://www.youtube.com/watch?v=MU0nSa5f8WQ&t=6s

© 2022 Goshgarian G et al. *JAMA Network Open.*

eTable 1. Logistic Regression Model of CRC Screening by Study Group

Effect	OR (95% CI)	P
Intervention v. Control	1.29 (1.08, 1.53)	**0.004**
Age (+1)	1.02 (1.01, 1.04)	**<0.001**
Male v. Female	1.15 (0.97, 1.37)	0.112
Ethnicity		
Non-Hispanic	1 [Reference]	1 [Reference]
Hispanic	0.86 (0.66, 1.13)	0.290
Unknown	1.59 (1.06, 2.40)	**0.026**
Race		
White	1 [Reference]	1 [Reference]
Black	1.04 (0.76, 1.43)	0.797
Asian	1.33 (1.02, 1.74)	**0.038**
American Indian or Alaska Native	<0.01 (<0.01, >99)	0.971
Other	1.04 (0.79, 1.35)	0.793
Unknown	0.56 (0.35, 0.91)	**0.018**

***Significance set at p < 0.05**

© 2022 Goshgarian G et al. *JAMA Network Open.*

eTable 2. Screening Modality by Study Group

	FOBT	Colonoscopy	Sigmoidoscopy	P
Intervention	409 (91.9%)	35 (7.9%)	1 (0.2%)	0.653
Control	337 (90.8%)	34 (9.2%)	0	

*These messages and material are proprietary information, and intellectual property of UCLA Health. Any, and all attempts to reproduce this information without the expressed consent of UCLA Health would be a violation, and could lead to legal ramifications.

© 2022 Goshgarian G et al. *JAMA Network Open.*
