# Supplementary material for: Effect of Patient Portal Messaging Before Mailing Fecal Immunochemical Test Kit on Colorectal Cancer Screening Rates: A Randomized Clinical Trial
Source: JAMA Netw Open. 2022 Feb 4;5(2):e2146863. doi: 10.1001/jamanetworkopen.2021.46863 (PMC8817202; doi:10.1001/jamanetworkopen.2021.46863)
Supplement: Supplement 3. — Data Sharing Statement [file jamanetwopen-e2146863-s003.pdf]

Data Sharing Statement

Goshgarian. Effect of Patient Portal Messaging Before Mailing Fecal Immunochemical Test Kit on Colorectal Cancer Screening Rates. *JAMA Netw Open*. Published February 04, 2022. doi:10.1001/jamanetworkopen.2021.46863

Data
Data available: No

Additional Information
Explanation for why data not available: The RCTs was pre-registered at clinicaltrials.gov (https://clinicaltrials.gov/ct2/show/NCT05115916). The data analysed in this Article about randomized controlled trials were provided by UCLA Health and contain protected health information. To protect participant privacy, we cannot publicly post individual-level data. Qualified researchers with a valuable research question and relevant approvals including ethical approval can request access to the de-identified data about these trials from the corresponding author. A formal contract will be signed and an independent data protection agency should oversee the sharing process to ensure the safety of the data.
